# The impact of smoking in the home on the health outcomes of non-smoker occupants in the UK

**DOI:** 10.1186/1617-9625-11-3

**Published:** 2013-01-29

**Authors:** Jeanette Kusel, Beth Timm, Ian Lockhart

**Affiliations:** 1 Costello Medical Consulting Ltd, St John’s Innovation Centre, Cowley Road, Cambridge, CB4 0WS, UK; 2 Pfizer Ltd, Walton Oaks, Dorking Road, Walton-on-the-Hill, Tadworth, KT20 7NS, Surrey, UK

**Keywords:** Second-hand smoking, Environmental tobacco smoke, Systematic literature review

## Abstract

Smoking in the home remains a key source of exposure to secondhand smoke for non-smokers, particularly since the UK public smoking ban in 2007. A systematic literature review was conducted to identify all UK evidence on the impact of secondhand smoke exposure in the home on health and behavioural outcomes in non-smoker occupants. MEDLINE, EMBASE and the Cochrane Library were searched to identify all relevant UK empirical studies from 2000 to June 2011. A qualitative overview of the evidence is presented. Exposure to secondhand smoke in UK homes was found to be associated with serious negative health effects in non-smokers, including significantly increased risk of meningococcal carriage (p < 0.001) and disease (p = 0.05) in children and adolescents, cognitive impairment (p < 0.001) in adults, a higher rate of medically attended accidents in children with smoking mothers (p < 0.01), and for non-smoking women, a significant decrease in infant birth weight (p = 0.007). Living in a smoking household significantly increased the risk of future regular smoking in children (p < 0.001). In conclusion, this systematic review has identified strong evidence of an association between secondhand smoke exposure in the home and several serious health conditions. This finding highlights the importance of educating current smokers on the consequences of non-smoker exposure to smoking in the home.

## Introduction

Secondhand smoke (SHS) is the smoke from the burning tip of a cigarette or exhaled smoke that is then inhaled by non-smokers. On a global scale, SHS was responsible for an estimated 600,000 deaths of non-smokers in 2011, the majority of which were women and children [1]. SHS exposure has been hailed as one of the world’s most critical environmental health hazards and there is no reported safe level of exposure [1]. SHS is known to be associated with an increased risk of smoking-related diseases such as lung cancer, heart disease, respiratory infection and meningitis [2-6].

In the UK, 21% of the population were smoking in 2010 [7]. The smoking ban in the UK has successfully reduced smoking in public places, work places, restaurants and bars, with some evidence of an improvement in health outcomes [8-10]. However, non-smokers living in smoking households continue to be exposed to high levels of SHS in the home and therefore passive exposure remains a major public health issue, particularly for children [11,12]. In 2007, 22% of children aged 4–15 in the UK lived in a home where someone smoked indoors [12]. Where one parent smoked, this parent did so inside the home in 63% of cases and where both parents smoked at least one of them did so inside the home in 79% of cases [12].

The main objective of this review was to collate the current UK evidence on the impact of exposure to SHS in the home on health outcomes of non-smoker occupants. Within this main objective, the review was divided into two distinct areas with the aim of assessing: (1) health outcomes of non-smokers previously and currently exposed to SHS in the home; and (2) risk of current or future smoking in children exposed to second hand smoke in the home.

## Methods

### Systematic review methods

A systematic and comprehensive literature search was conducted to identify all empirical studies that considered adverse events associated with exposure to SHS in the home, including parental, carer and family smoking in the UK setting. Studies reporting on home smoking were obtained through text word searches relating to ‘house’, ‘home’ or ‘domestic’, and combined with the MeSH headings ‘Tobacco Smoke Pollution’, ‘Smoking’, ‘Smoking Cessation’ and ‘Tobacco Use Cessation’ and relevant text word searches, including search terms for smoking related toxins/particles. Using these search strategies, the MEDLINE (including MEDLINE In Process), EMBASE and Cochrane Library databases were searched to June 2011. Search results were restricted from the year 2000 to ensure that only contemporary data on exposure and morbidity were used to inform the review.

### Inclusion and exclusion criteria

A single assessor reviewed the titles and abstracts of all search results and identified empirical studies specifically addressing: (1) the impact of past or current SHS exposure in the home on the health outcomes of non-smokers; and (2) the impact of home exposure as a child on the risk of future smoking behaviour. Studies were limited to the UK only.

Studies of low quality were excluded; low quality was defined as a score of 3 or less on the Newcastle Ottawa scale or admission by the study authors that it was underpowered for the outcomes of interest. A second, independent assessor reviewed all studies deemed potentially relevant by the first reviewer and confirmed inclusion. Disagreement between the reviewers was resolved through arbitration by a third party.

The study characteristics, population demographic data and outcomes of interest were extracted systematically from each included study by one reviewer and verified by a second, independent reviewer. Due to the differing populations across included studies and variation in the outcomes extracted across the research objectives, it was not possible to combine the results using statistical analysis; a qualitative description of outcomes of interest is given in the results.

### Assessment of study quality

All studies that met the inclusion criteria were critically appraised using the Newcastle-Ottawa scale, as recommended by the Cochrane collaboration for use with non-randomised, empirical studies [13]. This scale assesses the methodological quality of studies based on three categories: selection of the cohort of interest, comparability of the cohorts, and assessment of the outcomes of interest (for cohort studies) or the assessment of exposure (for case–control studies). The optimal assessment of exposure to SHS was considered to be measurement of cotinine levels in blood or saliva, as patient and parent-reported measures of SHS exposure often underestimate the extent of exposure, due to inaccurate reporting of smoking behaviour [14,15].

## Results

**Figure 1:**
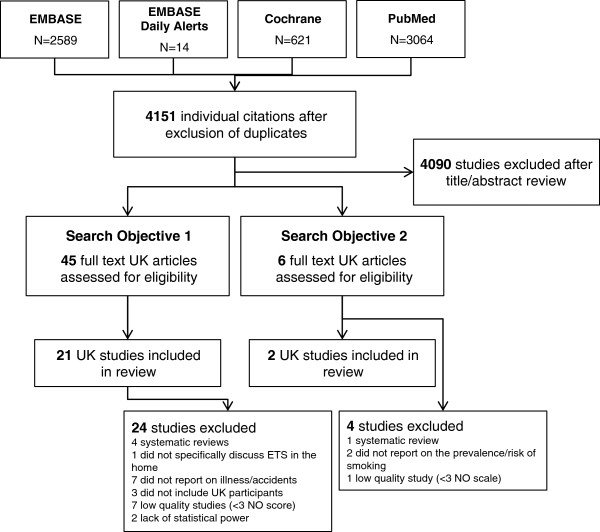
Flow diagram of included and excluded studies.

The literature searches identified 4151 individual citations once duplicates were removed, and of these 4090 were excluded on title or abstract. After full-text review of the remaining 51 articles, 33 were found to be relevant to the review objectives but 10 of these were excluded for low quality or lack of power; therefore 23 UK studies were included in the review (Figure 1).

The key characteristics for all 23 included studies pertaining to all search objectives are given in Table 1. Further details of the 10 studies excluded for their low quality or lack of statistical power are given in the Additional file 1: Table S1.

**Table 1:** Overview of the main characteristics for UK studies that report evidence on the impact of SHS exposure in the home on health and behavioural outcomes in non-smoker occupants

**Study ID**	**Type of Study**	**Included participants**	**Patient population**	**Assessment of SHS Exposure**	**Disease/Key Outcome**	**N-O Score**
***Health outcomes of non***-***smokers previously exposed to SHS in the home ***(***Objective 1***)						
**Reading et al. 2008**[16]	Longitudinal cohort	11,332 families	Families recruited in the ASLPAC study	Parent-completed postal questionnaire	Accidents	6
**Isle of Wight Birth Cohort**	Longitudinal cohort	1456	Birth cohort	Parent-completed questionnaire	Asthma/wheeze outcomes	7
**Arshad et al. 2005**[17]		1,373	Completed 10 year assessment			6
**Kurukulaaratchy et al. 2003**[18]		169	Positive for bronchial hyper-responsiveness at age 10			6
**Kurukulaaratchy et al. 2004**[19]		206	Early or late onset persistent wheeze at age 10			6
**Kurukulaaratchy et al. 2006**[20]		340	Reported wheeze ever up to age 10 with atopic parents			6
**Tariq et al. 2000**[21]		1,218	Completed 4 year assessment			7
**Hennessy et al. 2008**[22]	Longitudinal cohort	283	Babies born at 25 weeks gestation or less	Parent-completed questionnaire	Wheeze	7
**Murray et al. 2004**[23]	Longitudinal cohort	369	Child (<3 yrs) of parents with high risk of atopy	Interviewer administered respiratory questionnaire	Wheeze	6
**Trinder et al. 2000**[24]	Cross-sectional survey	2996	Adults (16+ yrs)	Patient-completed questionnaire	Respiratory symptoms	5
**Chen et al. 2001**[25]	Cross sectional survey	301	Never smoker adults (25–64 yrs)	Patient-completed questionnaire	Lung Function	4
**Palmer et al. 2004**[26]	Cross-sectional study	499	Child (3–21 yrs) diagnosed with asthma	Parent-completed questionnaire	Asthma	4
**Gee et al. 2005**[27]	Case–control study	95 controls	Child (4–16 yrs)	Air sampling (RSPs, tobacco specific particles, VOCs, NO2, formaldehyde)	Asthma	5
		105 cases				
**Forbes et al. 2007**[28]	Case–control study	394 controls	Child (3–14 yrs)	Parent-completed questionnaire	Asthma; Accident & Emergency attendance	5
		1018 cases				
**Crombie et al. 2001**[29]	Cross-sectional study	501 families/	Child (2–12 yrs) diagnosed with asthma	Saliva cotinine assessment; parent-completed questionnaire	Asthma	4
		438 children				
**Ward et al. 2007**[30]	Longitudinal retrospective cohort	16,756 parents	Neonates	Parent interview	Birth weight	7
**Macdonald Wallis et al. 2011**[31]	Longitudinal cohort	7121	Birth cohort	Parent-completed questionnaire	Bone characteristics	8
**Roddam et al. 2007**[32]	Case–control study	640 controls	Woman (aged 36–45 years) diagnosed with breast cancer from 1987-1990	Patient interview	Breast cancer	4
		639 cases				
**Llewellyn et al. 2009**[33]	Cross-sectional survey	4809	Non-smoking adults (>50 years)	Saliva cotinine	Cognitive impairment	5
**Williams et al. 2000**[34]	Cross-sectional survey	763	Child (1.5-4.5 years)	Parent-completed questionnaire	Dental caries	4
**MacLennan et al. 2006**[35]	Longitudinal cohort	13919	Adolescent (15–19 years)	Patient interview	Meningococcal carriage	6
**Coen et al. 2006**[36]	Case–control study	144 survivors 144 matched controls	Adolescent (15–19 yrs)	Patient interview	Meningococcal disease	4
***Risk of future smoking in children exposed to second hand smoke in the home ***(***Objective 2***)						
**Milton et al. 2004**[37]	Longitudinal cohort	247 at aged 9	Primary school children aged 9-11	Both child and parent (usually mother) postal questionnaires	Smoking behaviour assessed by self-completed questionnaire	9
		257 at aged				
		10				
		239 at aged 11				
**Griesbach et al. 2003**[38]	Cross-sectional survey	3132	Adolescents aged 15+ from Scotland (N = 1724) and Wales (N = 1408)	Self-completed questionnaire on parents and other smokers in the home	Smoking behaviour assessed by self-completed questionnaire	4

N-O Score, Newcastle-Ottawa score(Wells et al.). A higher score indicates higher methodological quality; max. 9.

RSP, respirable suspended particle; VOC, volatile organic compound.

### The impact of past and current exposure to secondhand smoke in the home

There were 21 UK studies of sufficient quality that assessed risk of illness or accidents related to past or current exposure to SHS in the home. Of these, 10 were longitudinal cohort, 4 were case control, and 7 were cross-sectional studies. Methodological details of the studies are presented in Table 1. All longitudinal studies scored 6 or above out of a possible 9 on the Newcastle-Ottawa scale. The highest degree of variability for all types of study was the lack of appropriate measure of SHS exposure [16,28,32,35,36]. The results are described below in alphabetical order of health condition.

#### Accidents

A large longitudinal cohort study of 11,332 children that followed them from birth to age 5 identified a significantly higher risk of accidents in children whose mothers smoked over the 5 year time period, for all accidents and medically attended accidents (Table 2) [16].

**Table 2:** Health outcomes of non-smokers exposed to SHS in the home

**Study ID**	**N**	**Outcome**		**OR/RR for SHS exposure compared to non-exposure**	**P value**
*Accidents*					
**Reading et al. 2008**[16]	11,332 families	For maternal smoking vs. non-smoking:			
		All accidents		RR 1.17, 95% CI 1.12-1.23	<0.01
		Medically attended accidents		RR 1.23, 95% CI 1.14-1.32	<0.01
*Asthma and Related Symptoms*					
***Isle of Wight Birth Cohort***					
**Arshad et al. 2005**[17]	1,373	Asthma at age 10 by parental smoking at age 1 vs. non-smoking		OR 1.99; 95% CI 1.15-3.45,	0.014
		Wheeze at age 10 by parental smoking at age 4 vs. non-smoking		OR 2.18; 95% CI 1.25-3.81	0.006
**Kurukulaaratchy et al. 2003**[18]	169	Bronchial hyper-responsiveness at 10 years by parental smoking at age 4 vs. non-smoking		OR 2.62, 95% CI 1.03-6.71	0.04
**Kurukulaaratchy et al. 2004**[19]	206	Early-onset persistent wheeze at age 10 vs. no parental smoking:	by parental smoking at birth	OR 1.88, 95% CI 1.27-2.77	0.001
			by parental smoking at 1 year	OR 2.05, 95% CI 1.39-3.02	<0.001
			by parental smoking at 2 years	OR 2.00, 95% CI 1.33-3.00	0.001
			by parental smoking at 4 years	OR 2.25, 95% CI 1.52-3.32	<0.001
**Kurukulaaratchy et al. 2006**[20]	340	Wheeze ever vs. no parental smoking	by parental smoking at 1 years	OR 1.54, 95% CI 1.07-2.22	0.02
			by parental smoking at 2 years	OR 1.51, 95% CI 1.04-2.21	0.03
			by parental smoking at 4 years	OR 1.80, 95% CI 1.25-2.58	0.001
**Tariq et al. 2000**[21]	1,218	Asthma at 4 years by exposure to SHS in the home in early childhood vs. no exposure		OR 1.2, 95% CI 0.3-2.7	NR
		Any allergic hypersensitivity at 4 years by exposure to SHS in the home in early childhood vs. no exposure		OR 1.1, 95% CI 0.6-1.6	NR
***Other studies***					
**Hennessy et al. 2008**[22]	219	Any wheeze at 6 years by exposure at 30 months vs. no exposure		OR 2.04, 95% CI 1.10-3.81	0.024
	218	Exercise-induced wheeze at 6 years by exposure at 30 months vs. no exposure		OR 2.14, 95% CI 1.11-4.12	0.022
	219	Night cough at 6 years by exposure at 30 months vs. no exposure)		OR 1.62, 95% CI 0.91-2.87	0.098
**Murray et al. 2004**[23]	369	For mother smoking postnatally vs. non-smoking			
			Wheeze ever	OR 1.93, 95% CI 1.10-3.38 (adjusted analysis)	0.02
			Wheeze in first year	OR 1.79, 95% CI 1.05-3.08 (adjusted analysis)	0.03
**Trinder et al. 2000**[24]	2996	Severe respiratory symptoms		OR 1.4, 95% CI 1.0-1.8	NR
**Chen et al. 2001**[25]	301	Lung function: mean residuals of FEV_1_ and FVC		NR	>0.05
**Palmer et al. 2006**[26]	504	Lung function		NR	>0.05
**Gee et al. 2005**[27]	95 controls	Difference in indoor pollutant levels between asthma cases and controls		NR	>0.05 for all pollutants
	105 cases				
**Forbes et al. 2007**[28]	394 controls	A&E attendance in asthma patients		OR 1.12, 95% CI 0.80-1.58 (adjusted analysis)	NR
	1018 cases				
**Crombie et al. 2001**[29]	501 families	Health service contacts for asthma by number of cigarettes smoked by parent per day (compared to 0–5)	6-10	IRR 0.81, 95% CI 0.71-0.92	0.0002 for trend
	438 children		11-15	IRR 0.70, 95% CI 0.59-0.83	
			16-20	IRR 0.74, 95% CI 0.61-0.91	
			>20	IRR 0.66, 95% CI 0.47-0.93	
*Birth weight*					
**Ward et al. 2007**[30]	16,756 parents	Mean birth weight (kg) difference between SHS exposed and non-exposed non smoking mothers	crude	NR	<0.001
			adjusted		0.025
*Bone Characteristics*					
**Macdonald Wallis et al. 2011**[31]	3591	TBLH bone area in girls at age 10 by paternal smoking during pregnancy vs. no smoking		NA	0.029 (fully adjusted analysis)
*Breast Cancer*					
**Roddam et al. 2007**[32]	640 controls, 639 cases	Breast cancer in never smokers		RR 0.89, 95% CI 0.64-1.25	NR
*Cognitive Impairment*					
**Llewellyn et al. 2009**[33]	4809	Cognitive impairment by cotinine level quartile in non-smoker (compared to lowest quartile):			
			Second quartile cotinine level	OR 1.08, 95% CI 0.78-1.48	0.02 for trend
			Third quartile cotinine level	OR 1.13, 95% CI 0.81-1.56	
			Fourth quartile cotinine level	OR 1.44, 95% CI 1.07-1.94	
*Dental Caries*					
**Williams et al. 2000**[34]	763	Dental caries			
			Non-manual occupations (n = 458)	OR 1.96, 1.00–3.85	0.05
			Manual occupations (n = 280)	OR 1.55, 1.02–2.35	<0.05
*Meningitis Carriage and Disease*					
**MacLennan et al. 2006**[35]	13,919	Meningococcal carriage in exposed (n = 5064)		OR 1.17, 95% CI 1.05-1.30	0.004
		vs. non-exposed (n = 8547)			
**Coen 2006**[36]	144 survivors	Meningococcal disease in adolescents exposed to smokers		OR 1.83; 95% CI 1.0–3.3	0.01
	144 matched controls				

A&E, accident and emergency; CI, confidence interval; IFEV, forced expiratory volume; FVC, forced vital capacity; RR, incidence rate ratio; NA, not appropriate; NR, not reported; OR, odds ratio; RR, risk ratio; TBLH, total body less head.

#### Asthma and respiratory symptoms

Eleven studies were included that reported on the association of SHS exposure (past or current) with asthma and other respiratory symptoms. Two additional studies reported on the association of exposure to SHS with the use of the healthcare system by asthmatic children.

Of the 6 studies identified that reported on non-smokers who have been exposed to SHS in the home in the past, 5 reported results from the Isle of Wight birth cohort study. The Isle of Wight birth cohort study was a prospective study to indentify risk factors relevant to wheezing and asthma. Of 1536 children born on the Isle of Wight between January 1st 1989 and February 28th 1990, 1456 children were enrolled. Enrolment took place at birth with information on family history of allergies, household pets, smoking habits, birth weight and social class being recorded. The children were followed up at the ages of 1, 2, 4 and 10 years [17-21]. In the sub-study of all children included at the 10 year assessment, parental smoking at age 1 was significantly associated with currently diagnosed asthma at age 10 (OR = 1.99 [95% CI 1.15-3.45]; p = 0.014) and parental smoking at age 4 was found to be significantly associated with current wheeze at age 10 (OR = 2.18 [95% CI 1.25-3.81]; p = 0.006; Table 2) [17]. Parental smoking at birth, 1 year, 2 years and 4 years was significantly associated with early-onset persistent wheeze (Table 2) [19,20]. Symptom expression in bronchial hyper-responsiveness, a hallmark of asthma, at 10 years was also significantly associated with parental smoking at age 4 (Table 2) [18]. However, no significant association was found between exposure to SHS in the home in early childhood (birth to 4 years) and respiratory allergic outcomes at 4 years old (Table 2) [21]. In a separate prospective cohort study, maternal smoking in the home at 30 months was associated with a significantly increased likelihood of any wheeze (OR = 2.04 [95% CI 1.10-3.81]; p = 0.024) and exercise-induced wheeze (OR = 2.14 [95% CI 1.11-4.12]; p = 0.022), but not night cough (OR 1.62 [95% CI 0.91-2.87]; p = 0.098), at 6 years (Table 2) [22]. These studies indicate how past smoking behaviour can be associated with future poor respiratory health.

Five studies were identified that reported on the association between current exposure to SHS in the home and asthma or respiratory symptoms. However, it should be noted that causality between current exposure and respiratory symptoms cannot be shown, as prior exposure and other factors may have influenced the health outcomes. In a cohort of high risk children where both parents demonstrated a predisposition toward developing allergic hypersensitivity there were increased odds of reporting wheeze ever and in the first year of life in children whose mother had smoked postnatally (wheeze ever: OR = 1.93 [95% CI 1.10-3.38]; p = 0.02, wheeze in first year of life: OR = 1.79 [95% CI 1.05-3.08]; p = 0.03; Table 2) [23]. In a large cross-sectional survey of a random sample of adults, those exposed to SHS in the home were significantly more likely to report severe respiratory symptoms compared to those with no exposure [24], whilst two other cross-sectional studies also showed a non-significant decrease in lung function associated with an increase in exposure to SHS in the home (Table 2) [25,26]. However, in a case control study that examined the difference in concentrations of indoor pollutants in the homes of children with asthma (cases) and those without (controls), no significant difference in concentrations of any indoor pollutant, including SHS specific particles, was found between asthma case and control environments (Table 2) [27].

In a case control study, no association was found between asthma patients’ Accident and Emergency (A&E) attendance and exposure to SHS in UK homes [28]. However, another cross-sectional study found that in asthmatic children, a reduction in the rate of health service contacts was significantly associated with increased number of cigarettes smoked in the home (Table 2) [29]. The explanation given by the study authors for the observed fewer health service contacts was that heavy smoking may reduce the awareness of parents to the child’s asthmatic symptoms.

#### Birth outcomes

One large retrospective birth cohort study demonstrated that for SHS exposed non-smoking mothers, there was a significant decrease in the crude and adjusted mean birth weight of their offspring compared to non SHS exposed mothers (crude mean difference: -0.059 kg, p < 0.001; adjusted mean difference: -0.036 kg, p = 0.025) [30]. Exposure to SHS was also associated with non-significant increases in the incidence of premature births (Table 2).

#### Bone characteristics

A longitudinal birth cohort study found that paternal smoking during pregnancy at weeks 18 and 32 was associated with a significant increase in total body-less-head bone area in girls but not boys at age 10 (Table 2) [31]. The main conclusion made by the authors was that the significant influence that paternal smoking factors had on girls’ health outcomes, combined with non-significant maternal smoking outcomes, could indicate that these associations are largely driven by familial characteristics related to childhood, and are unlikely to be due to intrauterine mechanisms.

#### Breast cancer

A UK case–control study of women aged 36–45 years who were diagnosed with invasive breast cancer reported no increased risk of breast cancer associated with exposure to SHS in the home through partner smoking (Table 2) [32].

#### Cognitive impairment

A large, cross-sectional study conducted in a cohort of non-smoking adults over 50 years of age identified a statistically significant increased odds of cognitive impairment associated with rising saliva cotinine levels in non-smokers (p = 0.02 for trend; OR = 1.44; Table 2) [33].

#### Dental caries

A cross-sectional survey demonstrated a significant increase in the odds of caries in pre-school children who had a smoking mother and were from families who had manual occupations (Table 2) [34].

#### Meningitis carriage and disease

Two studies, one case control and one longitudinal, were identified that reported an association between SHS exposure in the home and a significantly increased likelihood of meningococcal carriage and disease in adolescents, respectively [35,36]. Exposure to other smokers in the home was a significant factor for a positive test for meningococcal carriage according to a multivariable analysis (Table 2) [35]. Exposure to smokers was also found to be significantly and independently associated with meningococcal disease (Table 2), but this association is likely to be due to higher carriage rates in smokers rather than SHS exposure [36].

### The impact of secondhand smoke exposure on smoking behaviour

#### Summary of relevant studies

Only 2 UK studies were identified for inclusion that discussed the impact of exposure as a child on the risk of current or future smoking behaviour [37,38]. Of these studies, 1 was longitudinal, which scored the maximum score of 9 on the Newcastle-Ottawa scale, the other was a cross-sectional cohort study scoring 4. The key outcomes assessed were different frequencies of smoking: ever having tried smoking or daily smoking [37,38].

#### Impact on smoking behaviour

A longitudinal study found that significantly more children aged 10 and 11 had tried smoking if they lived with a smoker (p < 0.001; Table 3) compared to those who lived in a non-smoking household [37]. There was also a significant association between having tried smoking and living with a mother or sibling who smokes (Table 3) [37]. Exposure to SHS was not, however, found to be significantly associated with smoking behaviour [37]. A cross-sectional survey in Scotland and Wales also found that the presence of a parent smoker or other smoker was associated with a significantly increased likelihood of being a daily smoker compared with neither parent smoking or living with no smokers (p < 0.001 for all analyses; Table 3) [38].

**Table 3:** Risk of future smoking in children exposed to second hand smoke in the home

**Study ID**	**Behaviour outcome**	**N**	**OR for exposure compared to non-exposure**	**P value**
**Milton et al. 2004**[37]	Ever tried smoking:			
	Currently exposed to smokers in the home vs.	254 at aged 10	NR	<0.001
	non-smokers	238 at aged 11		<0.001
	Currently exposed to SHS in the home vs. not	256 at aged 10	NR	0.136 (aged 10)
	exposed	236 at aged 11		0.064 (aged 11)
	Tried smoking by age 11:			
	Exposed to smoking father at age 9	NR	OR 5.27, 95% CI 2.18 – 12.74	0.002
	vs. non-smoking father			
	Exposed to smoking brother at age 9	NR	OR 5.32, 95% CI 1.36 – 21.18	0.017
	vs. non-smoking brother			
**Griesbach et al. 2003**[38]	Being a daily smoker by the presence of either one or both parents who smoke			
	Scotland	1635	OR 1.73, 95% CI 1.32-2.26	<0.001
	Wales	1364	OR 1.98, 95% CI 1.46-2.70	<0.001
	Being a daily smoker by the presence of other (non-parent) smoker at home			
	Scotland	1635	OR 2.43, 95% CI 1.84-3.22	<0.001
	Wales	1364	OR 2.11, 95% CI 1.47-3.02	<0.001

CI, confidence interval; NR, not reported; OR, odds ratio.

Regarding future smoking, a logistic regression analysis from the longitudinal study found that having a father or brother who smoked when aged 9 meant that the child was over 5 times more likely to try smoking by age 11 than those who did not have a smoking father or brother at age 9 (Table 3) [37].

## Discussion-conclusions

Empirical UK evidence identified in this review has demonstrated that there are significant negative health outcomes associated with SHS exposure in the home, such as asthma, meningococcal disease, cognitive impairment, and dental caries.

In addition to the potential negative impact on health outcomes, SHS exposure in the home also seems to be related to smoking behaviour, particularly in children and adolescents. A significant proportion of children and adolescents in the UK who live in smoking households have tried smoking themselves, and living in a smoking household has been shown to be associated with an increased likelihood of regular smoking in the future [37,38]. Interestingly, time spent in a ‘smoky environment’ was not associated with ever trying smoking, although the same study showed that living with a smoker significantly increased the probability of ever trying smoking [37]. This suggests that the influence of smoking in the home is not only related to SHS exposure in itself; even if smoking members of the house are careful to smoke out of the direct environment of the non-smoker, there may still be a social or familial influence that encourages children to take up smoking.

The strengths of this review include the comprehensive search strategy, systematic data extraction and robust quality assessment method employed. The limitation of the review to UK studies reduced heterogeneity between study populations, but may have meant that other potential risks associated with SHS exposure in the home that have been identified outside of the UK will have been missed. The generalisability of the review results to countries outside of the UK is also unclear, although it is likely that the results will be relevant to countries that have had a smoking ban in public places for a similar length of time as the UK. There are several other limitations to this review, particularly due to the varied design of the studies that were included; the studies were often not comparable in terms of measurement of exposure to SHS or definition of health outcomes. The quality of each study was individually assessed using the Newcastle-Ottawa scale, and conclusions of the review have been limited to statements based on a good quality evidence base. Due to the difficulty of proving a causal link between SHS and health outcomes, most of the studies that were included reported statistical associations.

This review was limited to second hand smoke exposure only, but in addition to second hand exposure to smoke in the air there is also a risk of ‘third hand’ exposure, which refers to the residual tobacco smoke particles that remain after a cigarette is extinguished [39]. Children are highly susceptible to the ingestion of these third hand smoke particles [40]. Research conducted in the USA has shown that in smoking households high levels of surface nicotine can persist for several months even when smoking has ceased [41,42].

In summary, a review of the UK data on the impact of exposure to SHS in the home on health outcomes of non-smoker occupants has demonstrated a significantly increased probability of many negative health outcomes. Furthermore, smoking in the home appears to influence the future smoking behaviours of non-smokers. It is unclear how knowledgeable smokers are on the effects of smoking in the home, but the implication of this review is that it is essential that people who currently smoke in their home, particularly those with children or with a pregnant partner, are made aware of the potential impact of their smoking behaviour on non-smokers. Current smokers should be given adequate support to assist them in stopping smoking and reducing exposure of non-smokers to SHS within their home, which could include both counselling and access to pharmacotherapy. The evidence from this review lends support to the rationale and objectives of campaigns such as The Smoke Outside campaign from Smoke Free South West [43] and the Take 7 Steps Out campaign in the North East of the UK [44], both of which aim to educate people on the potential dangers of smoking in the home and to provide encouragement for people to smoke outside.

## Abbreviations

A&E: Accident and emergency; CI: Confidence interval; FEV: Forced expiratory volume; FVC: Forced vital capacity; IRR: Incidence risk ratio; NICE: National institute for health and clinical excellence; N-O: Newcastle-Ottawa scale; OR: odds ratio; RR: Risk ratio; RSP: Respirable suspended particle; SHS: Secondhand smoke; TBLH: Total body less head; VOC: Volatile organic compound.

## Competing interests

Ian Lockhart is an employee of Pfizer Ltd. Jeanette Kusel is an employee of Costello Medical Consulting and Beth Timm was a former employee, who received funding from Pfizer Ltd. to conduct the review.

## Authors’ contributions

JK and BT conducted the systematic literature review and contributed to the writing of the manuscript. IL reviewed the systematic literature review methods and results and contributed to the writing of the manuscript. All authors read and approved the final manuscript.

## Supplementary Material

Additional file 1: Table S1 Overview of the main characteristics for additional studies that report UK evidence on the impact of SHS exposure in the home on health and behavioural outcomes in non-smoker occupants not discussed in the main body of the manuscript. Click here for file

## References

[B1] World Lung Foundation,American Cancer Society The Tobacco Atlas, Chapter: Secondhand Smoking 2012 [cited 2012 August]; Available from: http://www.tobaccoatlas.org/harm/secondhand_smoking/youth/.

[B2] Holliday JC Moore GF Moore LA Changes in child exposure to secondhand smoke after implementation of smoke-free legislation in Wales: a repeated cross-sectional study BMC Publ Health 2009 9 430 10.1186/1471-2458-9-430 PMC2789068 19930678

[B3] Huttunen R Heikkinen T Syrjanen J Smoking and the outcome of infection J Intern Med 2011 269 258 269 10.1111/j.1365-2796.2010.02332.x 21175903

[B4] Jones LL Hashim A McKeever T Cook DG Britton J Leonardi-Bee J Parental and household smoking and the increased risk of bronchitis, bronchiolitis and other lower respiratory infections in infancy: systematic review and meta-analysis Respir Res 2011 12 5 10.1186/1465-9921-12-5 21219618 PMC3022703

[B5] Rushton L Health impact of environmental tobacco smoke in the home Rev Environ Health 2004 19 291 309 15742675

[B6] Stayner L Bena J Sasco AJ Smith R Steenland K Kreuzer M Straif K Lung cancer risk and workplace exposure to environmental tobacco smoke Am J Public Health 2007 97 545 551 10.2105/AJPH.2004.061275 17267733 PMC1805004

[B7] Office of National Statistics General lifestyle survey overview; a report on the 2010 General Lifestyle Survey 2010 Available from: http://www.ons.gov.uk/ons/rel/ghs/general-lifestyle-survey/2010/index.html.

[B8] Callinan JE Clarke A Doherty K Kelleher C Legislative smoking bans for reducing secondhand smoke exposure, smoking prevalence and tobacco consumption Cochrane Database Syst Rev 2010 14 CD005992 20393945 10.1002/14651858.CD005992.pub2

[B9] Healthier Scotland Clearing The Air 2006 [cited 2012 June]; Available from: http://www.clearingtheairscotland.com/background/index.html.

[B10] Smokefree England 2007 [cited 2012 June]; Available from: http://collections.europarchive.org/tna/20110202220654/http://www.smokefreeengland.co.uk.

[B11] Haw SJ Gruer L Changes in exposure of adult non-smokers to secondhand smoke after implementation of smoke-free legislation in Scotland: national cross sectional survey BMJ 2007 335 549 10.1136/bmj.39315.670208.47 17827485 PMC1976488

[B12] Passive Smoking and Children A Report of the Tobacco Advisory Group of the Royal College of Physicians 2010 [cited 2012 April]; Available from: http://bookshop.rcplondon.ac.uk/contents/pub305-e37e88a5-4643-4402-9298-6936de103266.pdf.

[B13] Wells G Shea B O’Connell D Peterson J Welch V Losos M Tugwell P The Newcastle-Ottawa Scale (NOS) for assessing the quality of nonrandomised studies in meta-analyses 2011 [cited 2012 April]; Available from: http://www.ohri.ca/programs/clinical_epidemiology/oxford.asp.

[B14] de Lorenze G Kharrazi M Kaufman F Eskenazi B Bernert JT Exposure to environmental tobacco smoke in pregnant women: The association between self-report and serum cotinine Environ Res 2002 90 21 32 10.1006/enrs.2001.4380 12359187

[B15] de Chazeron I Llorca P Ughetto S Coudore F Boussiron D Perriot J Vendittelli F Sapin V Lemery D Occult maternal exposure to environmental tobacco smoke exposure Tob Control 2007 16 64 65 10.1136/tc.2006.018291 17297076 PMC2598453

[B16] Reading R Jones A Haynes R Daras K Emond A Individual factors explain neighbourhood variations in accidents to children under 5 years of age Soc Sci Med 2008 67 915 927 10.1016/j.socscimed.2008.05.018 18573579

[B17] Arshad SH Kurukulaaratchy RJ Fenn M Matthews S Early life risk factors for current wheeze, asthma, and bronchial hyperresponsiveness at 10 years of age Chest 2005 127 502 508 10.1378/chest.127.2.502 15705988

[B18] Kurukulaaratchy RJ Matthews S Waterhouse L Arshad SH Factors influencing symptom expression in children with bronchial hyperresponsiveness at 10 years of age J Allergy Clin Immunol 2003 112 311 316 10.1067/mai.2003.1623 12897736

[B19] Kurukulaaratchy RJ Matthews S Arshad SH Does environment mediate earlier onset of the persistent childhood asthma phenotype? Pediatrics 2004 113 345 350 10.1542/peds.113.2.345 14754947

[B20] Kurukulaaratchy RJ Matthews S Arshad SH Relationship between childhood atopy and wheeze: what mediates wheezing in atopic phenotypes? Ann Allergy Asthma Immunol 2006 97 84 91 10.1016/S1081-1206(10)61375-0 16892787

[B21] Tariq SM Hakim EA Matthews SM Arshad SH Influence of smoking on asthmatic symptoms and allergen sensitisation in early childhood Postgrad Med J 2000 76 694 699 10.1136/pmj.76.901.694 11060143 PMC1741811

[B22] Hennessy EM Bracewell MA Wood N Wolke D Costeloe K Gibson A Marlow N Respiratory health in pre-school and school age children following extremely preterm birth Arch Dis Child 2008 93 1037 1043 10.1136/adc.2008.140830 18562451

[B23] Murray CS Woodcock A Smillie FI Cain G Kissen P Custovic A Tobacco smoke exposure, wheeze, and atopy Pediatr Pulmonol 2004 37 492 498 10.1002/ppul.20019 15114549

[B24] Trinder PM Croft PR Lewis M Social class, smoking and the severity of respiratory symptoms in the general population J Epidemiol Community Health 2000 54 340 343 10.1136/jech.54.5.340 10814653 PMC1731680

[B25] Chen R Tunstall-Pedoe H Tavendale R Environmental tobacco smoke and lung function in employees who never smoked: the scottish MONICA study Occup Environ Med 2001 58 563 568 10.1136/oem.58.9.563 11511742 PMC1740185

[B26] Palmer CN Doney AS Lee SP Murrie I Ismail T Macgregor DF Mukhopadhyay S Glutathione S-transferase M1 and P1 genotype, passive smoking, and peak expiratory flow in asthma Pediatrics 2006 118 710 716 10.1542/peds.2005-3030 16882827

[B27] Gee IL Watson AFR Tavernier G Stewart LJ Fletcher G Niven RML Indoor air quality, environmental tobacco smoke and asthma: a case control study of asthma in a community population Indoor and Built Environment 2005 14 215 219 10.1177/1420326X05054288

[B28] Forbes L Harvey S Newson R Jarvis D Luczynska C Price J Burney P Risk factors for accident and emergency (A&E) attendance for asthma in inner city children Thorax 2007 62 855 860 10.1136/thx.2006.058362 17456503 PMC2094271

[B29] Crombie IK Wright A Irvine L Clark RA Slane PW Does passive smoking increase the frequency of health service contacts in children with asthma? Thorax 2001 56 9 12 10.1136/thorax.56.1.9 11120897 PMC1745908

[B30] Ward C Lewis S Coleman T Prevalence of maternal smoking and environmental tobacco smoke exposure during pregnancy and impact on birth weight: retrospective study using millennium cohort BMC Publ Health 2007 7 81 10.1186/1471-2458-7-81 PMC1884144 17506887

[B31] Macdonald-Wallis C Tobias JH Davey Smith G Lawlor DA Parental smoking during pregnancy and offspring bone mass at age 10 years: findings from a prospective birth cohort Osteoporos Int 2011 22 1809 1819 10.1007/s00198-010-1415-y 20967424 PMC3092913

[B32] Roddam AW Pirie K Pike MC Chilvers C Crossley B Hermon C McPherson K Peto J Vessey M Beral V Active and passive smoking and the risk of breast cancer in women aged 36–45 years: a population based case–control study in the UK Br J Cancer 2007 97 434 439 10.1038/sj.bjc.6603859 17579618 PMC2360334

[B33] Llewellyn DJ Lang IA Langa KM Naughton F Matthews FE Exposure to secondhand smoke and cognitive impairment in non-smokers: national cross sectional study with cotinine measurement BMJ 2009 338 b462 10.1136/bmj.b462 19213767 PMC2643443

[B34] Williams SA Kwan SY Parsons S Parental smoking practices and caries experience in pre-school children Caries Res 2000 34 117 122 10.1159/000016578 10773628

[B35] MacLennan J Kafatos G Neal K Andrews N Cameron JC Roberts R Evans MR Cann K Baxter DN Maiden MC Stuart JM Social behavior and meningococcal carriage in British teenagers Emerging Infectious Diseases Journal 2006 12 950 957 10.3201/eid1206.051297 PMC3373034 16707051

[B36] Coen PG Tully J Stuart JM Ashby D Viner RM Booy R Is it exposure to cigarette smoke or to smokers which increases the risk of meningococcal disease in teenagers? Int J Epidemiol 2006 35 330 336 16394119 10.1093/ije/dyi295

[B37] Milton B Cook PA Dugdill L Porcellato L Springett J Woods SE Why do primary school children smoke? A longitudinal analysis of predictors of smoking uptake during pre-adolescence Public Health 2004 118 247 255 10.1016/j.puhe.2003.10.006 15121433

[B38] Griesbach D Amos A Currie C Adolescent smoking and family structure in Europe Soc Sci Med 2003 56 41 52 10.1016/S0277-9536(02)00014-X 12435550

[B39] Kuschner WG Reddy S Mehrotra N Paintal HS Electronic cigarettes and thirdhand tobacco smoke: two emerging health care challenges for the primary care provider International Journal of General Medicine 2011 4 115 120 21475626 10.2147/IJGM.S16908 PMC3068875

[B40] Winickoff JP Friebely J Tanski SE Sherrod C Matt GE Hovell MF McMillen RC Beliefs about the health effects of “thirdhand” smoke and home smoking bans Pediatrics 2009 123 e74 e79 10.1542/peds.2008-2184 19117850 PMC3784302

[B41] Matt GE Quintana PJ Zakarian JM Fortmann AL Chatfield DA Hoh E Uribe AM Hovell MF When smokers move out and non-smokers move in: residential thirdhand smoke pollution and exposure Tob Control 2011 20 e1 21037269 10.1136/tc.2010.037382 PMC3666918

[B42] Matt GE Quintana PJE Hovell MF Bernert JT Song S Novianti N Juarez T Floro J Gehrman C Garcia M Larson S Households contaminated by environmental tobacco smoke: Sources of infant exposures 2004 [cited 13 (Matt) Department of Psychology, San Diego State University, San Diego, CA 92182–4611, United States]; 1:[29-37]. 10.1136/tc.2003.003889 PMC1747815 14985592

[B43] Smoke Outside Campaign. Details available at http://smokeoutside.co.uk.

[B44] Take 7 Steps Out Campaign. Details available at http://www.take7stepsout.co.uk/.

