# Supplementary material for: The impact of smoking in the home on the health outcomes of non-smoker occupants in the UK
Source: Tob Induc Dis. 2013 Jan 29;11(1):3. doi: 10.1186/1617-9625-11-3 (PMC3568010; doi:10.1186/1617-9625-11-3)
Supplement: Additional file 1: Table S1 — Overview of the main characteristics for additional studies that report UK evidence on the impact of SHS exposure in the home on health and behavioural outcomes in non-smoker occupants not discussed in the main body of the manuscript. [file 1617-9625-11-3-S1.docx]

##### Supplementary Information:

Table S-1: Overview of the main characteristics for additional studies that report UK evidence on the impact of SHS exposure in the home on health and behavioural outcomes in non-smoker occupants not discussed in the manuscript.

| **Study ID** | **Type of Study** | **Included participants** | **Patient population** | **Assessment of ETS Exposure** | **Disease/Key Outcome** | **N-O Score** | **Reason for exclusion** |
| --- | --- | --- | --- | --- | --- | --- | --- |
| ***Health outcomes of non-smokers currently and previously exposed to ETS in the home*** | | | | | | | |
| **Tagiyeva et al. 2008** | Cross-sectional survey | 3271 | Child (7-12 yrs) | Parent-completed questionnaire | Wheezing illness | 3 | Low quality (N-O score of 3) |
| **Tavernier et al. 2006** | Case-control study | 200 | Child (4-17 yrs) diagnosed with asthma | Air sampling of tobacco specific particles as total respirable suspended particles | Asthma | 3 | Low quality (N-O score of 3) |
| **Austin et al. 2005** | Cross-sectional | 4665 | Child (13-14 yrs) diagnosed with asthma | Self-completed questionnaire | Asthma; Accident & Emergency attendance | 3 | Low quality (N-O score of 3) |
| **Lewis et al. 2005** | Cross-sectional survey | 11,562 | Child (4-6 yrs) | Parent-completed questionnaire | Wheeze | 3 | Low quality (N-O score of 3) |
| **Smyth et al. 2001** | Longitudinal cohort study | 108 | Children with cystic fibrosis | Parent-completed questionnaire, urinary and salivary cotinine | Cystic fibrosis | 6 | Underpowered (225 patients in each group would have been required for 90% power and 5% significance |
| **Spencer et al. 2003** | Cross-sectional/ longitudinal | 2,576 | Child (from birth) | Parent interview | Longstanding health problems | 3 | Low quality (N-O score of 3) |
| **Hepworth et al. 2010** | Cross-sectional survey | 10,032 | Child (6 months) | Parent-completed questionnaire | Infectious symptom profile | 3 | Low quality (N-O score of 3) |
| **Osman et al. 2007** | Cross-sectional survey | 206 | Patient admitted for exacerbation of COPD symptoms | Patient-completed questionnaire | COPD | 3 | Low quality (N-O score of 3) |
| **Venn et al. 2003** | Case-control study | 223 controls 193 cases | Child (6-8 yrs) | Saliva cotinine assessment; parent-completed questionnaire | Wheeze | 5 | Underpowered (250 patients in each group would have been required for 80% power and 5% significance |
| *Risk of future smoking in children exposed to second hand smoke in the home* | | | | | | | |
| **Withers et al. 2000** | Cross-sectional survey | 2150 | Adolescents aged 14 to 16 years old | Postal questionnaires sent to parents | Smoking behaviour assessed by self-completed postal questionnaire | 3 | Low quality (N-O score of 3) |
| ETS, Environmental tobacco smoke; DoH, Deportment of Health; RSP, Respirable suspended particle; VOC, Volatile organic compound | | | | | | | |
